# Variation in population levels of sedentary time in European children and adolescents according to cross-European studies: a systematic literature review within DEDIPAC

**DOI:** 10.1186/s12966-016-0395-5

**Published:** 2016-06-28

**Authors:** Maïté Verloigne, Anne Loyen, Linde Van Hecke, Jeroen Lakerveld, Ingrid Hendriksen, Ilse De Bourdheaudhuij, Benedicte Deforche, Alan Donnelly, Ulf Ekelund, Johannes Brug, Hidde P. van der Ploeg

**Affiliations:** Department of Movement and Sports Sciences, Faculty of Medicine and Health Sciences, Ghent University, Watersportlaan 2, 9000 Ghent, Belgium; Department of Epidemiology and Biostatistics, VU University Medical Center, EMGO+ Institute for Health and Care Research, De Boelelaan 1089a, 1081 HV Amsterdam, The Netherlands; Department of Public Health, Faculty of Medicine and Health Sciences, Ghent University, De Pintelaan 185, 9000 Ghent, Belgium; Physical activity, Nutrition and Health Research Unit, Department of Movement and Sport Sciences, Faculty of Physical Education and Physical Therapy, Vrije Universiteit Brussel, Pleinlaan 2, 1050 Brussels, Belgium; TNO Expertise Centre Lifestyle, Schipholweg 77-89, 2316 ZL Leiden, The Netherlands; Body@Work, EMGO+ Institute for Health and Care Research, VU University Medical Center, van der Boechorststraat 7, 1081 BT Amsterdam, The Netherlands; Centre for Physical Activity and Health Research, Department of Physical Education and Sport Sciences, University of Limerick, Limerick, Ireland; Department of Sports Medicine, Norwegian School of Sport Sciences, PO Box 4014, 0806 Ullevål Stadion, Oslo Norway; Department of Public and Occupational Health, VU University Medical Center, EMGO Institute for Health and Care Research, van der Boechorststraat 7, 1081 BT Amsterdam, The Netherlands; Sydney School of Public Health, The Charles Perkins Centre (D17), University of Sydney, NSW 2006 Sydney, Australia

**Keywords:** Youth, Prevalence, Assessment method, Health behaviour, Europe

## Abstract

**Background:**

A high amount of sedentary time has been proposed as a risk factor for various health outcomes in adults. While the evidence is less clear in children and adolescents, monitoring sedentary time is important to understand the prevalence rates and how this behaviour varies over time and by place. This systematic literature review aims to provide an overview of existing cross-European studies on sedentary time in children (0-12y) and adolescents (13-18y), to describe the variation in population levels of sedentary time, and to discuss the impact of assessment methods.

**Methods:**

Six literature databases were searched (PubMed, EMBASE, CINAHL, PsycINFO, SportDiscus and OpenGrey), followed by backward- and forward tracking and searching authors’ and experts’ literature databases. Included articles were observational studies reporting on levels of sedentary time in the general population of children and/or adolescents in at least two European countries. Population levels were reported separately for children and adolescents. Data were reviewed, extracted and assessed by two researchers, with disagreements being resolved by a third researcher. The review protocol is published under registration number CRD42014013379 in the PROSPERO database.

**Results:**

Forty-two eligible articles were identified, most were cross-sectional (*n* = 38). The number of included European countries per article ranged from 2 to 36. Levels of sedentary time were observed to be higher in East-European countries compared to the rest of Europe. There was a large variation in assessment methods and reported outcome variables. The majority of articles used a child-specific questionnaire (60 %). Other methods included accelerometers, parental questionnaires or interviews and ecological momentary assessment tools. Television time was reported as outcome variable in 57 % of included articles (ranging from a mean value of 1 h to 2.7 h in children and 1.3 h to 4.4 h in adolescents), total sedentary time in 24 % (ranging from a mean value of 192 min to 552 min in children and from 268 min to 506 min in adolescents).

**Conclusion:**

A substantial number of published studies report on levels of sedentary time in children and adolescents across European countries, but there was a large variation in assessment methods. Questionnaires (child specific) were used most often, but they mostly measured specific screen-based activities and did not assess total sedentary time. There is a need for harmonisation and standardisation of objective and subjective methods to assess sedentary time in children and adolescents to enable comparison across countries.

**Electronic supplementary material:**

The online version of this article (doi:10.1186/s12966-016-0395-5) contains supplementary material, which is available to authorized users.

## Background

Sedentary behaviour is defined as “any waking behaviour characterised by an energy expenditure of ≤ 1.5 metabolic equivalents while in a sitting or reclining position” [1]. The time spent in those sedentary behaviours has been defined as sedentary time. Although there is debate on the association between sedentary time and health outcomes in adults [2, 3], there are several studies, systematic reviews and meta-analyses showing that sedentary time has been positively associated with type 2 diabetes, cardiovascular diseases, metabolic syndrome and all-cause mortality among adults, independently from moderate to vigorous physical activity or subcomponents of physical activity [4–9]. Among children and adolescents, the evidence is less conclusive [10–12]. A possible reason is that some of the health outcomes may not be easily manifested in childhood or adolescence [10]. However, a recent review of reviews has suggested that there is an association between children’s screen-time behaviours (i.e. domain-specific sedentary behaviours) and obesity, blood pressure, total cholesterol, self-esteem, social behaviour problems, physical fitness and academic achievement [4]. Moreover, since sedentary time in early life may track into adulthood where it may have potential health implications and since children and adolescents spend a lot of time sedentary [13], actions may be considered to reduce time spent sedentary in children and adolescents. An important step to guide targeted action is to monitor the levels of sedentary time among children and adolescents across countries. This step is needed to study how the mean population levels of sedentary time vary by place, how it changes over time, and to evaluate preventive strategies and policies. In addition, it would be relevant to study and monitor the population levels of sedentary time specifically in Europe as it has its own governing structures but also a wide range of different cultures. Although the countries within Europe are diverse regarding political, economic, (socio-)cultural and physical environmental contexts, they are currently all struggling with an alarming increase in lifestyle related diseases such as overweight and obesity. This means that more effective efforts to reduce sedentary time in Europe are needed and monitoring the behaviour is a first step to address this need [14]. Focusing on specific European evidence is important to formulate public health guidelines and policy recommendations at the appropriate European level.

The DEDIPAC (DEterminants of DIet and Physical ACtivity) Knowledge Hub was established in 2013 by twelve European Union Member States [14]. One of the aims of DEDIPAC is “to enable a better standardised and more continuous cross-European monitoring of behaviours (including sedentary time) and changes in these behaviours across the life course and within populations to identify both targets and target populations for (policy) interventions”. A first and crucial step within DEDIPAC towards standardisation and harmonisation is to provide an overview of existing cross-European surveillance studies in order to describe population levels of (un)healthy behaviour by conjointly performing four systematic literature reviews. The reason to focus on cross-European studies is based on a 2010 WHO report concluding that even though population levels of health behaviour are frequently monitored across Europe, national surveys are not comparable due to differences in assessment methods [15]. Thus, focusing on cross-European initiatives at least enables within-study country comparison.

Therefore, this systematic literature review aims (a) to provide an overview of the existing cross-European studies (including data of at least two European countries) on sedentary time in children, (b) to describe the variation in population levels of sedentary time in European children and adolescents (0-18 years) according to these studies, and (c) to discuss the impact of assessment methods used. The other three reviews focus on the population levels of (1) sedentary time in adults [16], (2) physical activity in adults [17], and (3) physical activity in youth [18].

## Methods

As described in the introduction, this systematic literature review is part of a set of four reviews. Because the four systematic reviews originate from the same project, have similar objectives (although for different behaviours and/or age groups) and share their methodology, the introduction, methods and discussion sections of the review articles have obvious similarities. The search, article selection, data extraction and quality assessment were conducted conjointly for all four reviews. Subsequently, the included articles were allocated to the appropriate review article(s). If an article included both youth (<18 years) and adults (≥18 years) and presented stratified results, those stratified results were used in the appropriate review. If the article did not present stratified results, the article was allocated to the most appropriate review, based on the mean age (and age distribution) of the study sample. One article could be included in multiple reviews. Before the search commenced, review protocols were written based on the “Centre for Reviews and Dissemination’s guidance for undertaking reviews in health care” [19], and registered in the PROSPERO database (http://www.crd.york.ac.uk/PROSPERO/). The review protocol on sedentary time in youth is published under registration number CRD42014013379. The reporting of this systematic review adheres to the preferred reporting items of the PRISMA-P checklist (see Additional file 1).

### Search strategy

The search was conducted in June 2014 and updated on the 29^th^ of February, 2016. Six databases (PubMed, EMBASE, CINAHL, PsycINFO, SportDiscus and OpenGrey) were searched using similar search strategies, adapted to each database. The following search terms were used: ‘Physical activity’ OR ‘Sedentary behaviour’ AND ‘Europe’ (including all individual country names) AND ‘Countries‘/’Multicountry’/’International’. Both the index terms and the title and abstract were searched and synonyms (e.g. for sedentary behaviour: sitting, screen time, etc.) were used. The complete search string can be found in Additional file 2. Based on the in- and exclusion criteria described below, search filters of the databases were used when possible, for example to select the appropriate publication period or language. In addition, complementary search strategies were used. After the full-text review phase, the reference lists of the included articles were scanned (backward tracking) and a citation search was performed for the included articles (forward tracking) to identify potentially appropriate articles. Also, several experts in the field of physical activity and sedentary time were contacted to provide additional articles. Finally, all authors involved in the four reviews were asked to search their own literature databases for appropriate articles. All additionally retrieved articles underwent the same selection process as the original articles - as described below.

### Article selection

All retrieved records were imported into Reference Manager 12 (Thomson Reuters, New York). Duplicates were hand-searched and removed. Records were included if they were journal articles, reports or doctoral dissertations (further referred to as ‘articles’) written in English. To be included, articles needed to report on observational studies conducted after 01-01-2000 in the general, healthy population. This was done to avoid the reporting of outdated data. In addition, articles were only included if they provided data for two or more European countries (as defined by the Council of Europe) [20]. Articles were included if they reported total sedentary time (e.g. minutes/day), time spent sitting at school, time spent on screen-time behaviours (e.g. television viewing, using a computer) and/or time spent at any other sedentary activity. Both subjective (e.g. questionnaires) and objective (e.g. accelerometers) measures were included.

Three researchers (AL, LVH, MV) were involved in the article selection, data extraction and quality assessment. For the title selection, the three researchers each independently reviewed 1/3 of the titles of the retrieved articles. For the abstract and the full-text selection, data extraction and quality assessment, the three researchers each covered 2/3 of the articles, so that each article was independently reviewed, extracted and assessed by two different researchers. Disagreement between the two researchers was resolved by the third researcher.

### Data extraction

A standardised data extraction file was used to extract data regarding the study characteristics, study sample, assessment methods, reported outcomes, and findings. We did not obtain the original data. The complete data extraction file can be found in Additional file 3. To present the data more clearly and to allow for comparisons between age groups, the results are presented and discussed separately for children (aged 0-12 years) and adolescents (aged 13-18 years).

### Quality assessment

A quality score was used to provide a general overview of the quality of the included articles. The ‘Standard quality assessment criteria for evaluating primary research papers from a variety of fields’ was used for the assessment [21]. The checklist consists of fourteen items to be scored ‘Yes’ (2 points), ‘Partial’ (1 point), ‘No’ (0 points) and ‘Not applicable’. The summary score was calculated as follows: Total sum ((number of ‘Yes’ x 2) + (number of ‘Partial’ x 1))/Total possible sum (28 – (number of ‘Not applicable’ x 2)). This instrument was chosen because it provides the opportunity to assess and compare the quality of different study designs, focuses on both the research and the reporting, and allows researchers to indicate that an item is not applicable, without affecting the total quality score. The complete quality assessment file can be found in Additional file 4.

## Results

### Overview of the existing cross-European studies on sedentary time in children

**Fig. 1:**
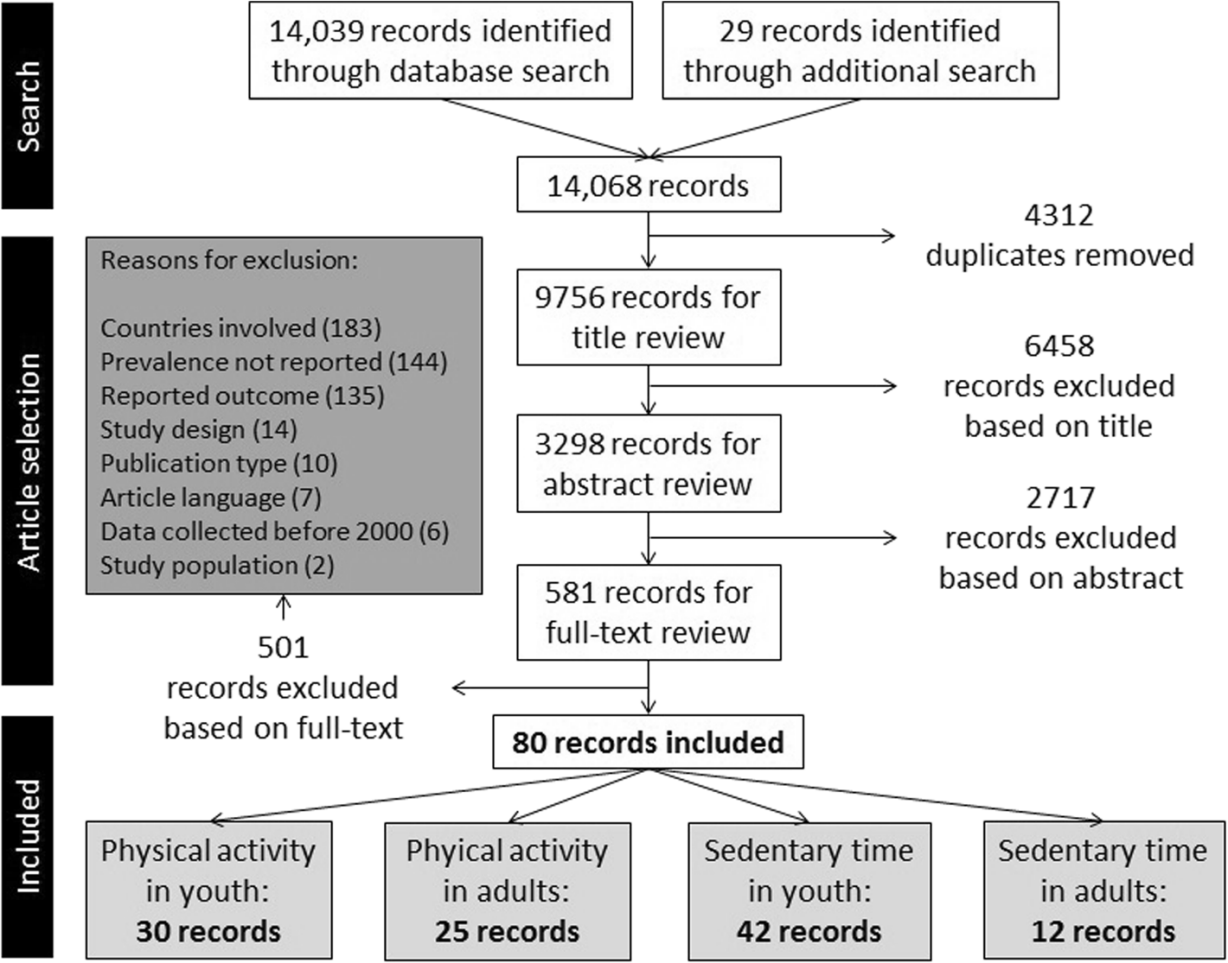
Flowchart of the combined review process

The search resulted in 9756 articles, after duplicates were removed. Based on titles and abstracts, the full text of 581 potentially relevant articles was retrieved and reviewed. This resulted in a total of 80 articles, of which 42 reported on levels of sedentary time in children and/or adolescents (Fig. 1) [22–63]. Table 1 provides an overview of the characteristics of the included articles. In brief, most articles were cross-sectional (*n* = 38), the quality score ranged from 0.64 to 1.0 on a scale from 0 to 1, the number of included European countries ranged from 2 to 36, and sample size ranged from 503 to 443,821. The majority of articles (*n* = 37) were part of a larger European study, that is the COSI study (1 article), ENERGY (6 articles), EYHS (5 articles), HBSC 01/02 (5 articles), HBSC 05/06 (3 articles), HBSC 09/10 (2 articles), HBSC 13/14 (1 article), ICAD (3 articles), IDEFICS (3 articles), ISAAC (1 article), ISCOLE (2 articles), Pro Children (2 articles), and Toybox (2 articles). One study reported data of HBSC 01/02, 05/06 and 09/10 together [49], which makes it possible to look at trends in sedentary time over time. Therefore, to describe the variation in population levels of sedentary time, we did not include all articles. If there was more than one article within a larger study reporting exactly the same outcome variable in a similar way in the same sample, only one article was included. These studies included data of all European countries, except for Andorra, Azerbaijan, Bosnia and Herzegovina, Georgia, Liechtenstein, Monaco, Montenegro, San Marino and Serbia.

**Table 1:** Study information and sample characteristics of the articles included in the systematic review

Publication	Study	Study design	Quality score (0-1)	Number of EU countries	Number of EU partici-pants	Demographics				Sedentary time assessment method	Reported sedentary time variable
						Age range	Gender, girls	SES	Weight status		
**Biddle et al. (2009)** ^**a**^ **[** 22 **]**	/	CS	0.91	3	623	13-18y	60.4 %	15.0 - 36.1 % low SES	n.r.	Ecological Momentary Assessment diary	min/weekday and min/weekend day technical sedentary behaviours, social sedentary behaviours
Soos et al. (2012) [23]	/	CS	0.83	2	635	13.1-18.0y	60.5 %	n.r.	n.r.	Ecological Momentary Assessment diary	min/day television viewing, doing homework, motorised transport, sitting and talking, computer use, reading, sitting doing nothing, videogames
**Soos et al. (2014) [** 24 **]**	/	CS	0.86	4	700	11.9-17.9y	57 %	n.r.	n.r.	Ecological Momentary Assessment diary	min/day television viewing, computer use, playing computer games, telephone use, motorised transport, sitting and talking, doing homework, reading
**Cinar & Murtomaa (2008)** ^**a**^ **[** 25 **]**	/	CS	0.77	2	619	10-12y	43.9 - 49.1 %	n.r.	18.7 kg/m^2^	Child questionnaire	% favorable: <2 h/day television viewing % unfavorable: >2 h/day television viewing
**Hanewinkel et al. (2012) [** 26 **]**	/	CS	0.95	6	16551	10-19y	49 %	10 % low SES	n.r.	Child questionnaire	h/schoolday television viewing: % None, % less than 1 h, % 1–2 h, % 3–4 h, % >4 h
**Börnhorst et al. (2015) [** 27 **]**	COSI	CS	0.95	5	10453	6.0-9.9y	49.4 %	16.5 % par. Master’s degree or higher	26.9 % over-weight	Child questionnaire	h/day television time, computer time, screen-time
**Brug et al. (2012)** ^**a**^ **[** 28 **]**	ENERGY	CS	1.00	7	7234	10-12y	52 %	15.7-48.4 % low par. edu.	18.1 - 20.6 kg/m^2^	Child questionnaire	min/day screen-time, television viewing and computer use (FQ and 24 h-recall)
Brug et al. (2012)^a^ [29]	ENERGY	CS	0.91	7	7307	10-12y	52 %	29-59 % low par. edu.	19.0-19.5 kg/m^2^	Child questionnaire	min/day screen-time
Fernandez-Alvira et al. (2013) [30]	ENERGY	CS	0.95	7	5284	10-12y	54.3 %	32.5 % low par. edu.	20.4 % over-weight	Child questionnaire	min/day screen-time
**van Stralen et al. (2014) [** 31 **]**	ENERGY	CS	0.95	5	1025	10-12y	51 %	45 % low par. edu.	19.0 kg/m^2^	ActiGraph accelerometer	min/school-time sedentary time + percentage of total school-time spent in sedentary activities
**Verloigne et al. (2012) [** 32 **]**	ENERGY	CS	0.95	5	687	10-12y	53 %	n.r.	19.0 kg/m^2^	ActiGraph accelerometer	min/day sedentary time
Yildirim et al. (2014) [33]	ENERGY	CS	0.95	5	722	10-12y	53 %	14 % not speaking native language at home	n.r.	ActiGraph accelerometer	min/day sedentary time
Ekelund et al. (2004) [34]	EYHS	CS	1.00	4	1292	9-10y	50.6 %	n.r.	17.2 kg/m^2^	MTI ActiGraph accelerometer	% sedentary activity per day
Jago et al. (2008) [35]	EYHS	CS	0.95	4	2670	9y and 15y	51.1 %	n.r.	13.1 % over-weight	Child questionnaire	% <2 h, % ≥2 h television viewing after school % <1 h, % ≥1 h/day computer use
**Nilsson et al. (2009)** ^**a**^ **[** 36 **]**	EYHS	CS	1.00	4	1954	9y and 15y	47.9 – 63.2 %	n.r.	n.r.	MTI ActiGraph accelerometer	min/weekday, min/weekend day, min/school-time, min/leisure-time sedentary time
**Ortega et al. (2013)** ^**a**^ **[** 37 **]**	EYHS	LT cohort	0.91	2	503	15y and 18y	55.4-56.7 %	27.6-33.3 % mother university (baseline)	16.4 – 17.3 kg/m^2^ (base-line)	ActiGraph accelerometer	min/day, weekday and weekend day sedentary time
**van Sluijs et al. (2008)** ^**a**^ **[** 38 **]**	EYHS	CS	0.95	4	2107	9y and 15y	43.9-54.4 %	6.7-10.8 mean edu./income (3-16)	18.1-19.2 kg/m^2^	Child questionnaire	% >1 h television before school % >2 h television after school % >1 h/day computer use
**Janssen et al. (2005)** ^**a**^ **[** 39 **]**	HBSC 01/02	CS	0.95	29	128845	10-16y	47.1 - 53.3 %	n.r.	5.1 - 25.4 % over-weight	Child questionnaire	% high television viewing = >3 h/weekday % high computer use = >2 h/weekday
Kuntsche et al. (2006) [40]	HBSC 01/02	CS	0.91	5	19877	11y, 13y, 15y	52.6 %	n.r.	n.r.	Child questionnaire	h/weekday and h/weekend day television viewing
Richter et al. (2009)^a^ [41]	HBSC 01/02	CS	0.95	24	76794	13y, 15y	52.2 %	22.7-41.9 % low FAS	n.r.	Child questionnaire	% ≥ 4 h/day television viewing
Vereecken et al. (2006) [42]	HBSC 01/02	CS	0.91	28	148150	11y, 13y, 15y	n.r.	n.r.	n.r.	Child questionnaire	h/day television viewing
HBSC report 2004^a^ [43]	HBSC 01/02	CS	0.73	28	146368	11y, 13y, 15y	51.5 %	27.6 % low FAS	7.1 – 12.1 % pre-obese	Child questionnaire	% ≥4 h/weekday and weekend day television viewing % ≥3 h/weekday and weekend day computer use % ≥3 h/weekday and weekend day homework
**Haug et al. (2009)** ^**a**^ **[** 44 **]**	HBSC 05/06	CS	1.00	34	187657	11y, 13y, 15y	49.3 %	n.r.	6.3 – 18.5 % pre-obese	Child questionnaire	% less than 2 h/day television viewing, computer games, computer use
Torsheim et al. (2010) [45]	HBSC 05/06	CS	0.91	5	31022 (all 6 countries)	11y, 13y, 15y	n.r.	n.r.	n.r.	Child questionnaire	h/day of computer use, computer games, television viewing
HBSC report 2008^a^ [46]	HBSC 05/06	CS	0.68	35	188147	11y, 13y, 15y	50.7 %	2-70 % low FAS	13-14 % over-weight	Child questionnaire	% ≥2 h/weekday television viewing, computer use, computer games/game console
Nuutinen et al. (2015) [47]	HBSC 09/10	CS	1.00	3	5402	15y	53 %	n.r.	n.r.	Child questionnaire	h:min/day computer use schooldays
HBSC report 2012^a^ [48]	HBSC 09/10	CS	0.68	35	178531	11y, 13y, 15y	51 %	2 %-42 % low FAS	10-18 % over-weight	Child questionnaire	% ≥ 2 h/weekday of television viewing
**Bucksch et al. (2016)** ^**a**^ **[** 49 **]**	HBSC 01/02, HBSC 05/06, HBSC 09/10	CS	0.82	24	443821 (total sample)	11y, 13y, 15y	51.2-51.4 %	n.r.	n.r.	Child questionnaire	h/weekday and weekend day television viewing, computer use
							(total sample)				
**HBSC report 2016** ^**a**^ **[** 50 **]**	HBSC 13/14	CS	0.64	36	199316	11y, 13y, 15y	50.7 %	38-76 FAS score (0-100)	15 % over-weight	Child questionnaire	% ≥ 2 h/weekday of television viewing % ≥ 2 h/weekday of computer use ≥2 h/weekday of playing games
**Atkin et al. (2014)** ^**a**^ **[** 51 **]**	ICAD	Pooled data (CS and LT)	0.82	5	5474	8-17y	48.9-56.7 %	4.8-52.6 % mother university	9.4-24.0 % over-weight	Child or parental questionnaire	% ≥ 2/day screen time
**Ekelund et al. (2012)** ^**a**^ **[** 52 **]**	ICAD	Pooled data (CS and LT)	0.91	7	15614	4-18y	51.6 %	n.r.	19.1-19.4 kg/m^2^	ActiGraph accelerometer	min/day sedentary time
Hildebrand et al. (2015) [53]	ICAD	Pooled data (CS and LT)	0.91	6	10367	6-18y	53 %	n.r.	15.9 % over-weight; 4.8 % obese	ActiGraph accelerometer	min/day sedentary time
**Hense et al. (2011) [** 54 **]**	IDEFICS	CS	0.91	8	8542	2-9y	49.2 %	27.2 % low SES	20.2 % over-weight	Parental questionnaire	h/day screen-time. % not at all, % <0.5 h, % 0.5-1 h, % 1-2 h, % 2-3 h, % >3 h
Hunsberger et al. (2012)^a^ [55]	IDEFICS	CS	0.86	8	12720	2-9y	47.7-51.4 %	1.2 – 30.8 % low edu. household	7.7 – 41.9 % over-weight	Parental questionnaire	% <1 h/day screen-time
Kovàcs et al. (2015) [56]	IDEFICS	CS	0.95	16	16228	2-9.9y	49.1 %	10.7 % low edu. level	Mean BMI z-score: 0.33	Parental questionnaire	% <1 h/day screen-time (pre-schoolers) % <2 h/day screen-time (school children)
**Mitchell et al. (2013) [** 57 **]**	ISAAC	CS	0.86	6-7y: 6	6-7ys: 33901	6-7y and 13-14y	n.r.	n.r.	n.r.	Child questionnaire	h/day television viewing: % < 1 h, % 1-3 h, % 3-5 h, % > 5 h
				13-14y: 7	13-14y: 61954						
Katzmaryk et al. (2015)^a^ [58]	ISCOLE	CS	0.95	3	1664	9-11y	53.8-55.9 %	n.r.	17.7-19.5 kg/m^2^	ActiGraph accelerometer	min/day sedentary time
**LeBlanc et al. (2015)** ^**a**^ **[** 59 **]**	ISCOLE	CS	0.95	3	1496	9-11y	53.1-57.2 %	21.1-73.2 % high par. edu.	24.3-45.7 % over-weight	ActiGraph accelerometer and child questionnaire	h/day sedentary time h/day screen-time % ≥ 2 h/day of screen-time
**Klepp et al. (2007)** ^**a**^ **[** 60 **]**	Pro Children	CS	1.00	9	12773	8.8-13.8y	49.8 %	71.6 - 82.1 % not in social class I-II	n.r.	Child questionnaire	h/day television viewing
**te Velde et al. (2007) [** 61 **]**	Pro Children	CS	0.95	9	12538	8.8-13.8y	50.1 %	n.r.	n.r.	Child questionnaire	% <2 h/day television viewing % >1 h/day computer use
**De Craemer et al. (2015) [** 62 **]**	Toybox	CS	0.95	6	8117	3.5-5.5y	47 %	n.r.	n.r.	Parental questionnaire	min/weekday and weekend day television viewing, computer use, quiet play % <1 h/day screen-time weekday and weekend day
**van Stralen et al. (2012)** ^a^ **[** 63 **]**	ToyBox	Pooled data (CS)	0.91	5	6097	4-7y	47.4 - 52.0 %	n.r.	15.9 – 16.8 kg/m^2^	Parental questionnaire	h/day television viewing, % ≥ 2 h/day of television viewing, h/day screen-time, min/day sedentary time (sedentary play-time + screen-time)

*COSI* WHO European Childhood Obesity Surveillance Initiative, *ENERGY* EuropeaN Energy balance Research to prevent excessive weight Gain among Youth, *EYHS* European Youth Heart Study, *HBSC* Health Behaviour in School-aged Children, *ICAD* International Children’s Accelerometer Database, *IDEFICS* Identification and prevention of Dietary and lifestyle induced health Effects In Children and infantS, *ISAAC* International Study of Asthma and Allergies in Childhood, *ISCOLE* The International Study of Childhood Obesity, Lifestyle and the Environment, *CS* cross-sectional, *LT* longitudinal, *n.r.* not reported, *SES* socio-economic status, *par. edu.* parental education, *inc.* income, *FAS* Family Affluence Scale, *FQ* frequency question, ^a^These articles only presented stratified demographics, so the range is reported; articles in bold were included in Tables 2 and 3

### Variation in population levels of sedentary time in European children and adolescents

The population levels of sedentary time in children (0-12y) and adolescents (13-18y) are presented by country in Tables 2 and 3, respectively. For this research question, 24 articles were included. In Table 1, these 24 studies are indicated in bold. The first column of both Tables 2 and 3 shows how the specific type of sedentary activity (e.g. total sedentary time, TV time) was reported (e.g. percentage or minutes) over a specific time period (e.g. weekend day, after school). To keep the Tables as comparable as possible, we only included values of the total sample, except if an article only reported results for boys and girls separately. Some articles also reported the outcome variable separately for regions within a country. For the HBSC-report that was released in 2016 with data of 2013/2014 [50], the values of the 11-year-olds were included in the Table for children, and the values of the 15-year-olds were included in the Table for adolescents.

**Table 2:** Levels of sedentary time in children (0-12 years) across European countries

**Total sedentary time**	**Armenia**	**Albania**		**Austria**	**Belgium**	**Bulgaria**	**Croatia**	**Cyprus**	**Czech Republic**
Min, h or %/day					478 min(B)^33^, 511 min(G)^33^, 232 min^64^				
Min or %/weekday									
Min or %/weekend day									
Min or %/school time					65 %(G)^32^, 61 %(B)^32^				
Min or %/leisure time									
**Television time**									
Min or h/day				2.2 h^61^	116 min(G)^29,FQ^, 110 min(B)^29,FQ^, 78 min(G)^29,recall^, 77 min(B)^29,recall^, 2.7 h^61^	1.8 h^28^, 1.8 h^64^			1.2 h^28^
Min or h/weekday					67 min^63^	79 min^63^			
Min or h/weekend day					116 min^63^	131 min^63^			
% >1 h before school									
% >2 h/day				36(B)^62^, 32(G)^62^	50(B)^62^, 42(G)^62^				
% >2 h/weekday	48(B)^51^, 47(G)^51^	51(B)^51^, 47(G)^51^		50(B)^51^, 40(G)^51^	55(B,FL)^51^, 54(G,FL)^51^, 48(B,FR)^51^, 43(G,FR)^51^	64(B)^51^, 66(G)^51^	49(B)^51^, 47(G)^51^		62(B)^51^, 48(G)^51^
% <1 h/day, 1-3 h/day, 3-5 h/day, >5 h/day									
**Computer time**									
Min or h/day					89 min(B)^29,FQ^, 69 min(G)^29,FQ^, 47 min(B)^29,recall^, 29 min(G)^29,recall^	0.7 h^28^			0.5 h^28^
Min or h/weekday					15 min^63^	28 min^63^			
Min or h/weekend day					29 min^63^	44 min^63^			
% >1 h/day				41(B)^62^, 16(G)^62^	35(B)^62^, 20(G)^62^				
% >2 h/weekday	27^51^	20^51^		26^51^	32(FL)^51^, 28(FR)^51^	50^51^	26^51^		35^51^
**Videogames time**									
% >2 h/weekday	23^51^	28^51^		31^51^	33(FL)^51^, 33(FR)^51^	56^51^	25^51^		37^51^
**Total screen-time**									
Min or h/day					205 min(B)^29,FQ^, 178 min(G)^29,FQ^, 124 min(B)^29,recall^, 107 min(G)^29,recall^	2.5 h^28^			1.7 h^28^
% <1 h/weekday					43^63^	25^63^			
% <1 h/weekend day					16^63^	9^63^			
% >2 h/day									
% not at all, <0.5 h, 0.5-1 h, 1-2 h, 2-3 h, >3 h/day					2, 13, 32, 28, 15, 11^55^			2, 8, 20, 32, 17, 12^55^	
**Total sedentary time**	**Denmark**			**Estonia**	**Finland**	**France**	**Germany**	**Greece**	**Hungary**
Min, h or %/day	268 min^53^, 356 min^53^			343 min^53^	8.8 h^60^			526 min(B)^33^, 510 min(G)^33^,	487 min(B)^33^, 475 min(G)^33^
Min or %/weekday	311 min(B)^37^, 309 min(G)^37^			277 min(B)^37^, 307 min(G)^37^					
Min or %/weekend day	299 min(B)^37^, 280 min(G)^37^			239 min(B)^37^, 257 min(G)^37^					
Min or %/school time	115 min(B)^37^, 128 min(G)^37^			122 min(B)^37^, 138 min(G)^37^				61 %(B)^32^, 66 %(G)^32^	65 %(B)^32^, 70 %(G)^32^
Min or %/leisure time	152 min(B)^37^, 136 min(G)^37^			132 min(B)^37^, 146 min(G)^37^					
**Television time**									
Min or h/day	2.2 h^61^							126 min(B)^29,FQ^, 120 min(G)^29,FQ^, 99 min(B)^29,recall^, 89 min(G)^29,recall^, 2.2 h^64^	123 min(B)^29,FQ^, 116 min(G)^29,FQ^, 90 min(B)^29,recall^, 85 min(G)^29, recall^
Min or h/weekday							43 min^63^	89 min^63^	
Min or h/weekend day							65 min^63^	134 min^63^	
% >1 h before school	4^39^			14^39^					
% >2 h/day	38(B)^62^, 32(G)^62^				15^26^				
% >2 h/weekday	15^39^, 60(B)^51^, 49(G)^51^			42^39^, 61(B)^51^, 56(G)^51^	58(B)^51^, 55(G)^51^	50(B)^51^, 39(G)^51^	45(B)^51^, 36(G)^51^	53(B)^51^, 45(G)^51^	47(B)^51^, 40(G)^51^
% <1 h/day, 1-3 h/day, 3-5 h/day, >5 h/day				9, 58, 24, 8^58^					17, 63, 14, 5^58^
**Computer time**									
Min or h/day								88 min(B)^29,FQ^, 60 min(G)^29,FQ^, 55 min(B)^29,recall^, 33 min(G)^29,recall^	110 min(B)^29,FQ^, 82 min(G)^29,FQ^, 75 min(B)^29,recall^, 46 min(G)^29,recall^
Min or h/weekday							9 min^63^	18 min^63^	
Min or h/weekend day							15 min^63^	30 min^63^	
% >1 h/day	15^39^, 39(B)^62^, 13(G)^62^			16^39^					
% >2 h/weekday	40^51^			37^51^	33^51^	29^51^	27^51^	25^51^	27^51^
**Videogames time**									
% >2 h/weekday	53^51^			43^51^	30^51^	33^51^	25^51^	28^51^	34^51^
**Total screen-time**									
Min or h/day					2.7^60^		0.7 h^64^	214 min(B)^29,FQ^, 179 min(G)^29,FQ^, 155 min(B)^29,recall^, 122 min(G)^29,recall^	233 min(B)^29,FQ^, 198 min(G)^29,FQ^, 166 min(B)^29,recall^, 131 min(G)^29,recall^
% <1 h/weekday							71^63^	29^63^	
% <1 h/weekend day							52^63^	12^63^	
% >2 h/day	34^52^, 47^52^			62^52^	57^60^				
% not at all, <0.5 h, 0.5-1 h, 1-2 h, 2-3 h, >3 h/day				1, 6, 19, 24, 18, 32^55^			4, 12, 26, 26, 14, 13^55^		4, 15, 27, 25, 15, 12^55^
**Total sedentary time**	**Iceland**	**Ireland**	**Italy**	**Latvia**	**Lithuania**	**Luxembourg**	**Malta**	**Moldova**	**Netherlands**
Min, h or %/day									447 min(B)^33^, 457 min(G)^33^
Min or %/weekday									
Min or %/weekend day									
Min or %/school time									65 %(B)^32^, 68 %(G)^32^
Min or %/leisure time									
**Television time**									
Min or h/day	2.0 h^61^				1.8 h^28^				116 min(B)^29, FQ^, 104 min(G)^29, FQ^, 83 min(B)^29, recall^, 67 min(G)^29, recall^, 2.7 h^61^
Min or h/weekday									
Min or h/weekend day									
% >1 h before school									
% >2 h/day	35(B)^62^, 23(G)^62^								50(B)^38^, 46(G)^38^
% >2 h/weekday	40(B)^51^, 30(G)^51^	46(B)^51^, 42(G)^51^	47(B)^51^, 40(G)^51^	63(B)^51^, 56(G)^51^	59(B)^51^, 54(G)^51^	44(B)^51^, 37(G)^51^	53(B)^41^, 41(G)^51^	54(B)^51^, 53(G)^51^	61(B)^51^, 61(G)^51^
% <1 h/day, 1-3 h/day, 3-5 h/day, >5 h/day					19, 64, 15, 2^58^				
**Computer time**									
Min or h/day					0.9 h^28^				106 min (B)^29,FQ^, 81 min (G)^29,FQ^, 71 min(B)^29,recall^, 45 min(G)^29,recall^
Min or h/weekday									
Min or h/weekend day									
% >1 h/day	36(B)^62^, 12(G)^62^								53(B)^62^, 26(G)^62^
% >2 h/weekday	31^51^	29^51^	27^51^	38^51^	26^51^	27^51^	35^51^	31^51^	42^51^
**Videogames time**									
% >2 h/weekday	39^51^	31^51^	32^51^	39^51^	40^51^	29^51^	42^51^	36^51^	49^51^
**Total screen-time**									
Min or h/day					2.6 h^28^				223 min(B)^29,FQ^, 185 min(G)^29,FQ^, 153 min(B)^29,recall^, 112 min(G)^29,recall^
% <1 h/weekday									
% <1 h/weekend day									
% >2 h/day									
% not at all, <0.5 h, 0.5-1 h, 1-2 h, 2-3 h, >3 h/day			2, 8, 20, 27, 19, 24^55^						
**Total sedentary time**	**Norway**	**Poland**		**Portugal**	**Romania**	**Russian Federation**		**Slovakia**	**Slovenia**
Min, h or %/day	325 min^53^			367 min^53^, 9.2 h^60^					
Min or %/weekday	298 min(B)^37^, 314 min(G)^37^			318 min(B)^37^, 344 min(G)^37^					
Min or %/weekend day	289 min(B)^37^, 280 min(G)^37^			269 min(B)^37^, 279 min(G)^37^					
Min or %/school time	128 min(B)^37^, 140 min(G)^37^			146 min(B)^37^, 153 min(G)^37^					
Min or %/leisure time	137 min(B)^37^, 138 min(G)^37^			153 min(B)^37^, 169 min(G)^37^					
**Television time**									
Min or h/day	105 min(B)^29,FQ^, 97 min(G)^29,FQ^, 72 min(B)^29,recall^, 62 min(G)^29,recall^, 2.2 h^61^			1.3 h^28^, 2.7 h^61^					120 min(B)^29,FQ^, 108 min(G)^29,FQ^, 78 min(B)^29,recall^, 68 min(G)^29,recall^
Min or h/weekday		71 min^63^							
Min or h/weekend day		116 min^73^							
% >1 h before school	9^39^			15^39^					
% >2 h/day	38(B)^62^, 35(G)^62^			49(B)^62^, 42(G)^62^					
% >2 h/weekday	25^39^, 46(B)^51^, 41(G)^51^	56(B)^51^, 49(G)^51^		31^39^, 52(B)^51^, 45(G)^51^	67(B)^51^, 56(G)^51^	57(B)^51^, 52(G)^51^		59(B)^51^, 54(G)^51^	49(B)^51^, 40(G)^51^
% <1 h/day, 1-3 h/day, 3-5 h/day, >5 h/day		18, 69, 11, 3(Krakow)^58^; 11, 73 12, 3(Poznan)^58^		13, 58, 23, 6^58^					
**Computer time**									
Min or h/day	91 min(B)^29,FQ^, 71 min(G)^29,FQ^, 60 min(B)^29,recall^, 40 min(G)^29,recall^			0.5 h^28^					93 min(B)^29,FQ^, 64 min(G)^29,FQ^, 52 min(B)^29,recall^, 33 min(G)^29,recall^
Min or h/weekday		16 min^63^							
Min or h/weekend day		32 min^63^							
% >1 h/day	27^39^, 24(B)^62^, 10(G)^62^			27^39^, 40(B)^62^, 17(G)^62^					
% >2 h/weekday	34^51^	35^51^		24^51^	35^51^	42^51^		40^51^	25^51^
**Videogames time**									
% >2 h/weekday	31^51^	33^51^		25^51^	44^51^	42^51^		43^51^	24^51^
**Total screen-time**									
Min or h/day	196 min(B)^29,FQ^, 168 min (G)^29,FQ^, 132 min (B)^29,recall^, 101 min (G)^29,recall^			1.8 h^28^, 2.3 h^60^					213 min(B)^29, FQ^, 174 min(G)^29, FQ^, 131 min(B)^29, recall^, 100 min(G)^29, recall^
% <1 h/weekday		37^63^							
% <1 h/weekend day		16^63^							
% >2 h/day	49^52^			64^52^, 49^60^					
% not at all, <0.5 h, 0.5-1 h, 1-2 h, 2-3 h, >3 h/day									
**Total sedentary time**	**Spain**	**Sweden**		**Switzerland**	**Macedonia**	**Turkey**		**Ukraine**	**UK**
Min, h or %/day				467 min(B)^33^, 498 min(G)^33^, 236 min^53^, 278 min^53^					356 min^53^, 362 min^53^, 352 min^53^, 192 min(SC)^53^, 8.3 h^60^
Min or %/weekday									
Min or %/weekend day									
Min or %/school time									
Min or %/leisure time									
**Television time**									
Min or h/day	109 min(B)^29,FQ^, 97 min(G)^29,FQ^, 77 min(B)^29,recall^, 64 min(G)^29,recall^, 2.2 h^61^	1.3 h^28^, 2.1 h^61^							
Min or h/weekday	66 min^63^								
Min or h/weekend day	122 min^63^								
% >1 h before school									
% >2 h/day	37(B)^62^, 31(G)^62^, 8^64^	32(B)^62^, 31(G)^62^				28^26^			
% >2 h/weekday	43(B)^51^, 30(G)^51^	58(B)^51^, 51(G)^51^		32(B)^51^, 29(G)^51^	46(B)^51^, 43(G)^51^			52(B)^51^, 46(G)^51^	51(B,ENG)^51^, 51(G,ENG)^51^, 60(B,SC)^51^, 51(G,SC)^51^, 62(B,WAL)^51^, 53(G,WAL)^51^
% <1 h/day, 1-3 h/day, 3-5 h/day, >5 h/day	24, 62, 12, 2(A Coruña)^58^; 27, 59, 11, 3(Asturias)^58^; 19, 59, 19, 3(Barcelona)^58^; 34, 54, 10, 2(Bilbao)^58^; 15, 63, 19, 4(Cartagena)^58^; 18, 61, 18, 3(Madrid)^58^; 22, 61, 14, 2(Valencia)^58^								
**Computer time**									
Min or h/day	85 min(B)^29,FQ^, 63 min(G)^29,FQ^, 45 min(B)^29,recall^, 25 min(G)^29,recall^	0.6 h^28^							
Min or h/weekday	13 min^63^								
Min or h/weekend day	31 min^63^								
% >1 h/day	22(B)^62^, 15(G)^62^	35(B)^62^, 18(G)^62^							
% >2 h/weekday	22^51^	40^51^		18^51^	36^51^			33^51^	
**Videogames time**									
% >2 h/weekday	23^51^	44^51^		20^51^	34^51^			33^51^	41(ENG)^51^, 51(SC)^51^, 49(WAL)^51^
**Total screen-time**									41(ENG)^51^, 44(SC)^51^, 50(WAL)^51^
Min or h/day	193 min(B)^29,FQ^, 160 min(G)^29,FQ^, 122 min(B)^29,recall^, 89 min(G)^29,recall^	1.9 h^28^							2.9 h^60^
% <1 h/weekday	44^63^								
% <1 h/weekend day	12^63^								
% >2 h/day									47(ENG)^52^, 59(ENG)^52^, 68^60^
% not at all, <0.5 h, 0.5-1 h, 1-2 h, 2-3 h, >3 h/day	6, 22, 28, 26, 12, 6^55^								

This table displays a summary of the results reported in the articles included in the systematic review; *B* boys, *G* girls, *min* minutes, *h* hours, *FQ* usual frequency question, *FL* Flemish part of Belgium, *FR* French part of Belgium, *ENG* England, *SC* Scotland, *WAL* Wales; references are displayed in superscript to avoid confusion with the levels of sedentary time

**Table 3:** Levels of sedentary time in adolescents (13-18 years) across European countries

**Total sedentary time**	**Albania**	**Armenia**	**Austria**	**Belgium**	**Bulgaria**	**Croatia**	**Czech Republic**
Min or %/day							
Min or %/weekday							
Min or %/weekend day							
Min or %/school time							
Min or %/leisure time							
**Television time**							
Min or h/day							
Min or h/weekday				2.6-2.5-2.3 h(B,FL)^50^, 2.3-2.4-2.2 h(G,FL)^50^, 2.2-2.2-2.0 h(B,FR)^50^, 2.1-1.9-1.8 h(G,FR)^50^		3.0-3.0-2.7 h(B)^50^, 2.7-2.8-2.6 h(G)^50^	2.8-2.5-2.3 h(B)^50^, 2.5-2.3-2.2 h(G)^50^
Min or h/weekend day				3.7-3.4-3.2 h(B,FL)^50^, 3.1-3.2-3.1 h(G,FL)^50^, 3.5-3.4-3.2(B,FR)^50^, 3.2-3.1-3.1 h(G,FR)^50^		3.9-3.5-3.3 h(B)^50^, 3.7-3.4-3.2 h(G)^50^	3.2-3.2-3.0 h(B)^50^, 2.9-2.9-2.7 h(G)^50^
% >2 h/day			38(B)^45^, 33(G)^45^	40(B,FL)^45^, 40(G,FL)^45^, 33(B,FR)^45^, 26(G,FR)^45^	60(B)^45^, 66(G)^45^	44(B)^45^, 50(G)^45^	42(B)^45^, 38(G)^45^
% >2 h/weekday	73(B)^51^, 75(G)^51^	73(B)^51^, 66(G)^51^	54(B)^51^, 57(G)^51^	61(B,FL)^51^, 59(G,FL)^51^, 64(B,FR)^51^, 55(G,FR)^51^	70(B)^51^, 72(G)^51^	66(B)^51^, 59(G)^51^	65(B)^51^, 59(G)^51^
% >3 h/weekday			31^40^	40(FL)^40^, 34(FR)^40^		53^40^	47^40^
% <1 h/day, 1-3 h/day, 3-5 h/day, >5 h/day				9, 39, 31, 20^58^			
% ≤0.5 h, 1-2 h, 3-4 h, >4 h/schoolday							
**Computer time**							
Min or h/day							
Min or h/weekday				1.4-3.3-3.2 h(B,FL)^50^, 0.9-2.5-2.5 h(G,FL)^50^, 1.4-2.9-2.8 h(B;FR)^50^, 0.7-2.4-2.4 h(G,FR)^50^		1.2-2.7-3.8 h(B)^50^, 0.5-1.6-2.9 h(G)^50^	1.6-3.2-4.0 h(B)^50^, 0.7-1.9-3.0 h(G)^50^
Min or h/weekend day				2.3-4.7-4.7 h(B,FL)^50^, 1.4-3.4-3.5 h(G,FL)^50^, 2.5-5.1-4.9 h(B,FR)^50^, 1.2-4.0-4.2 h(G,FR)^50^		1.9-3.7-5.0 h(B)^50^, 0.9-2.2-3.9 h(G)^50^	1.9-4.0-4.8 h(B)^50^, 0.8-2.4-3.6 h(G)^50^
% >1 h/day							
% >2 h/day			20(B)^45^, 15(G)^45^	23(B,FL)^45^, 23(G,FL)^45^, 17(B,FR)^45^, 15(G,FR)^45^	30(B)^45^, 24(G)^45^	12(B)^45^, 10(G)^45^	14(B)^45^, 14(G)^45^
% >2 h/weekday	50^51^	48^51^	25^40^, 53^51^	26(FL)^40^, 22(FR)^40^, 60(FL)^51^, 60(FR)^51^	69^51^	20^40^, 57^51^	26^40^, 65^51^
**Videogames time**							
Min or h/day							
% >2 h/day			28(B)^45^, 12(G)^45^	22(B,FL)^45^, 8(G,FL)^45^, 23(B,FR)^45^, 15(G,FR)^45^	44(B)^45^, 18(G)^45^	25(B)^45^, 5(G)^45^	31(B)^45^, 7(G)^45^
% >2 h/weekday	40^51^	35^51^	36^51^	32(FL)^51^, 49(FR)^51^	53^51^	32^51^	42^51^
**Total screen-time**							
Min or h/day							
Min or h/weekday							
**Total sedentary time**	**Denmark**		**Estonia**	**Finland**	**France**	**Germany**	**Greece**
Min or %/day	268 min^53^, 356 min^53^		506 min(B)^38^, 496 min(G)^38^, 343 min^53^				
Min or %/weekday	454 min(B)^37^, 457 min(G)^37^		388 min(B)^37^, 344 min(G)^37^, 526 min(B)^38^, 521 min(G)^38^				
Min or %/weekend day	412 min(B)^37^, 412 min(G)^37^		331 min(B)^37^, 367 min(G)^37^, 459 min(B)^38^, 434 min(G)^38^				
Min or %/school time	205 min(B)^37^, 218 min(G)^37^		186 min(B)^37^, 227 min(B)^37^				
Min or %/leisure time	205 min(B)^37^, 191 min(G)^37^		168 min(B)^37^, 187 min(B)^37^				
**Television time**							
Min or h/day							
Min or h/weekday	2.6-2.4-2.4 h(B)^50^, 2.5-2.3-2.2 h(G)^50^		3.4-2.8-2.4(B)^50^, 3.0-2.7-2.4 h(G)^50^	2.3-2.0-2.0 h(B)^50^, 2.3-1.9-1.8 h(G)^50^	2.3-2.3-2.1 h(B)^50^, 2.1-2.1-2.0 h(G)^50^	2.4-2.3-2.1 h(B)^50^, 2.2-2.1-2.0 h(G)^50^	2.5-2.7-2.7 h(B)^50^, 2.1-2.8-2.5 h(G)^50^
Min or h/weekend day	3.3-3.2-3.2 h(B)^50^, 3.1-3.0-3.0 h(G)^50^		4.0-3.5-3.3(B)^50^, 3.9-3.5-3.2 h(G)^50^	3.3-2.8-2.8 h(B)^50^, 3.1-2.7-2.7 h(G)^50^	3.2-3.2-3.0 h(B)^50^, 3.0-2.9-2.9 h(G)^50^	3.4-3.5-3.3 h(B)^50^, 3.0-3.1-3.0 h(G)^50^	3.5-3.6-3.6 h(B)^50^, 3.3-3.6-3.5 h(G)^50^
% >1 h before school	40(B)^45^, 36(G)^45^		51(B)^45^, 50(G)^45^	28(B)^45^, 27(G)^45^	37(B)^45^, 32(G)^45^	36(B)^45^, 33(G)^45^	48(B)^45^, 53(G)^45^
% >2 h/day							
% >2 h/weekday	71(B)^51^, 68(G)^51^		59(B)^51^, 57(G)^51^	61(B)^51^, 52(G)^51^	62(B)^51^, 58(G)^51^	66(B)^51^, 60(G)^51^	71(B)^51^, 65(G)^51^
% >3 h/weekday	45^40^		63^40^	40^40^	34^40^, 64^51^	39^40^	38^40^
% <1 h/day, 1-3 h/day, 3-5 h/day, >5 h/day			4, 28, 40, 28^58^	5, 39, 37, 19^58^			
% ≤0.5 h, 1-2 h, 3-4 h, >4 h/schoolday						25, 52, 17, 6^27^	
**Computer time**							
Min or h/day							
Min or h/weekday	1.9-3.6-4.3 h(B)^50^,		1.8-4.4-4.5 h(B)^50^,	1.4-3.2-3.4 h(B)^50^,	1.0-2.7-3.3 h(B)^50^,	1.5-3.2-3.2 h(B)^50^,	1.2-2.6-3.7 h(B)^50^,
	0.7-2.1-2.9 h(G)^50^		0.9-3.0-3.5 h(G)^50^	0.6-2.0-2.5 h(G)^50^	0.6-1.9-2.6 h(G)^50^	0.7-2.2-2.6 h(G)^50^	0.5-1.1-2.3 h(G)^50^
Min or h/weekend day	2.3-4.4-5.4 h(B)^50^,		2.3-5.5-5.7 h(B)^50^,	2.0-4.2-4.7 h(B)^50^,	1.5-4.1-4.9 h(B)^50^,	2.0-4.7-4.8 h(B)^50^,	1.9-4.2-5.5 h(B)^50^,
	0.8-2.5-3.4 h(G)^50^		1.1-3.8-4.4 h(G)^50^	0.8-2.7-3.5 h(G)^50^	0.9-2.9-3.9 h(G)^50^	1.0-3.0-3.7 h(G)^50^	0.8-1.8-3.7(G)^50^
% >1 h/day							
% >2 h/day	20(B)^45^, 17(G)^45^		32(B)^45^, 30(G)^45^	17(B)^45^, 17(G)^45^	16(B)^45^, 16(G)^45^	19(B)^45^, 17(G)^45^	10(B)^45^, 4(G)^45^
% >2 h/weekday	29^40^, 67^51^		32^40^, 72^51^	23^40^, 59^51^	16^40^	25^40^, 66^51^	21^40^, 59^51^
**Videogames time**							
Min or h/day							
% >2 h/day	32(B)^45^, 8(G)^45^		41(B)^45^, 14(G)^45^	24(B)^45^, 6(G)^45^	20(B)^45^, 6(G)^45^	25(B)^45^, 10(G)^45^	26(B)^45^, 6(G)^45^
% >2 h/weekday	42^51^		41^51^	29^51^	38^51^	50^51^	38^51^
**Total screen-time**							
Min or h/day							
Min or h/weekday							
**Total sedentary time**	**Hungary**		**Iceland**	**Ireland**	**Italy**	**Latvia**	**Lithuania**
Min or %/day							
Min or %/weekday							
Min or %/weekend day							
Min or %/school time							
Min or %/leisure time							
**Television time**							
Min or h/day	100 min^25^						
Min or h/weekday	2.4-2.3-2.2 h(B)^50^, 2.2-2.1-2.1 h(G)^50^				2.3-2.3-2.1 h(B)^50^, 2.5-2.2-2.0 h(G)^50^	3.4-3.0-2.5 h(B)^50^, 2.9-2.8-2.5 h(G)^50^	
Min or h/weekend day	4.0-3.7-3.6 h(B)^50^, 3.9-3.6-3.5 h(G)^50^				2.6-2.6-2.6 h(B)^50^, 2.6-2.4-2.4 h(G)^50^	4.4-3.6-3.2 h(B)^50^, 4.1-3.5-3.2 h(G)^50^	
% >2 h/day	40(B)^45^, 36(G)^45^		36(B)^45^, 29(G)^45^		36(B)^45^, 37(G)^45^		
% >2 h/weekday	62(B)^51^, 58(G)^51^		58(B)^51^, 54(G)^51^	56(B)^51^, 54(G)^51^	59(B)^51^, 52(G)^51^	68(B)^51^, 67(G)^51^	58(B)^51^, 58(G)^51^
% >3 h/weekday	39^40^			38^40^	43^40^	63^40^	57^40^
% <1 h/day, 1-3 h/day, 3-5 h/day, >5 h/day	7, 48, 32, 14^58^						12, 51, 26, 11^58^
% ≤0.5 h, 1-2 h, 3-4 h, >4 h/schoolday			29, 55, 13, 3^27^		20, 48, 23, 9^27^		
**Computer time**							
Min or h/day	8 min^25^						
Min or h/weekday	1.4-3.0-3.7 h(B)^50^, 0.7-1.8-2.6 h(G)^50^				1.1-2.3-3.1 h(B)^50^, 0.7-1.4-2.7 h(G)^50^	1.4-3.6-3.9 h(B)^50^, 0.7-2.4-2.8 h(G)^50^	
Min or h/weekend day	2.4-5.2-6.2 h(B)^50^, 1.2-3.2-4.5 h(G)^50^				1.3-2.7-3.8 h(B)^50^, 0.8-1.6-3.3 h(G)^50^	2.0-4.3-4.8 h(B)^50^, 1.0-2.9-3.5 h(G)^50^	
% >1 h/day							
% >2 h/day	18(B)^45^, 12(G)^45^		26(B)^45^, 23(G)^45^		9(B)^45^, 8(G)^45^		
% >2 h/weekday	23^40^, 58^51^		61^51^	61^51^	20^40^, 55^51^	27^40^, 65^51^	23^40^, 46^51^
**Videogames time**							
Min or h/day	26 min^25^						
% >2 h/day	24(B)^45^, 8(G)^45^		27(B)^45^, 3(G)^45^		17(B)^45^, 4(G)^45^		
% >2 h/weekday	44^51^		40^51^	30^51^	41^51^	39^51^	48^51^
**Total screen-time**							
Min or h/day	156 min(B)^23^, 114 min(G)^23^						
Min or h/weekday	282 min(B)^23^, 192 min(G)^23^						
**Total sedentary time**	**Luxembourg**	**Moldova**	**Malta**	**Netherlands**	**Norway**	**Poland**	**Portugal**
Min or %/day					325 min^55^		367 min^55^
Min or %/weekday					445 min(B)^37^, 466 min(G)^37^		411 min(B)^37^, 435 min(G)^37^
Min or %/weekend day					385 min(B)^37^, 402 min(G)^37^		344 min(B)^37^, 351 min(G)^37^
Min or %/school time					206 min(B)^37^, 228 min(G)^37^		206 min(B)^37^, 217 min(G)^37^
Min or %/leisure time					189 min(B)^37^, 190 min(G)^37^		183 min(B)^37^, 191 min(G)^37^
**Television time**							
Min or h/day							
Min or h/weekday				2.8-3.0-2.8 h(B)^50^, 2.4-2.7-2.6 h(G)^50^	2.7-2.1-2.0 h(B)^50^, 2.6-2.2-2.0 h(G)^50^	3.0-2.6-2.5 h(B)^50^, 2.6-2.4-2.3 h(G)^50^	2.8-3.0-2.5 h(B)^50^, 2.9-3.0-2.5 h(G)^50^
Min or h/weekend day				3.6-3.4-3.2 h(B)^50^, 3.3-3.3-3.1 h(G)^50^	3.6-3.0-2.9 h(B)^50^, 3.3-2.9-2.9 h(G)^50^	4.0-3.8-3.4 h(B)^50^, 3.7-3.7-3.4 h(G)^50^	3.9-4.0-3.8 h(B)^50^, 3.8-4.0-3.9 h(G)^50^
% >2 h/day	32(B)^45^, 28(G)^45^			50(B)^45^, 44(G)^45^		55(B)^45^, 41(G)^45^	54(B)^45^, 56(G)^45^
% >2 h/weekday	66(B)^51^, 61(G)^51^	73(B)^51^, 77(G)^51^	65(B)^51^, 54(G)^51^	73(B)^51^, 75(G)^51^	63(B)^51^, 61(G)^51^	62(B)^51^, 64(G)^51^	55(B)^51^, 51(G)^51^
% >3 h/weekday			43^40^	45^40^	48^40^	53^40^	52^40^
% <1 h/day, 1-3 h/day, 3-5 h/day, >5 h/day						9, 46, 29, 17(Krakow)^58^; 8, 54, 27, 12(Poznan)^58^	11, 37, 30, 22(Funchal)^58^; 5,36, 36, 23(Lisbon)^58^; 7, 36, 34, 23(Portimao)^58^; 8, 45, 30, 18(Porto)^58^
% ≤0.5 h, 1-2 h, 3-4 h, >4 h/schoolday				24, 57, 17, 2^27^		19, 49, 24, 8^27^	
**Computer time**							
Min or h/day							
Min or h/weekday				1.7-4.6-4.5 h(B)^50^,	1.9-3.1-3.3 h(B)^50^,	1.6-4.2-4.8 h(B)^50^,	1.5-3.8-3.8 h(B)^50^,
				1.0-3.2-3.4 h(G)^50^	0.8-2.2-2.5 h(G)^50^	0.8-2.2-3.2 h(G)^50^	0.7-2.6-2.8 h(G)^50^
Min or h/weekend day				2.4-5.1-4.9 h(B)^50^, 1.4-3.7-3.6 h(G)^50^	2.3-4.1-4.1 h(B)^50^, 1.0-2.8-2.8 h(G)^50^	2.5-6.0-6.5 h(B)^50^, 1.3-3.4-4.7 h(G)^50^	2.2-5.2-5.9 h(B)^50^, 1.0-3.4-4.3 h(G)^50^
% >1 h/day					26.8^12^		29.7^30^
% >2 h/day	18(B)^45^, 17(G)^45^		31(B)^45^, 31(G)^45^	36(B)^45^, 35(G)^45^		30(B)^45^, 21(G)^45^	23(B)^45^, 22(G)^45^
% >2 h/weekday	67^51^	66^51^	20^40^, 69^51^	31^40^, 78^51^	31^40^, 74^51^	32^40^, 70^51^	25^40^, 49^51^
**Videogames time**							
Min or h/day							
% >2 h/day	21(B)^45^, 9(G)^45^			37(B)^45^, 10(G)^45^		36(B)^45^, 8(G)^45^	36(B)^45^, 14(G)^45^
% >2 h/weekday	44^51^	41^51^	57^51^	56^51^	48^51^	32^51^	32^51^
**Total screen-time**							
Min or h/day							
Min or h/weekday							
**Total sedentary time**	**Romania**	**Russian Federation**	**Slovakia**		**Slovenia**	**Spain**	**Sweden**
Min or %/day							486 min(B)^38^, 482 min(G)^38^
Min or %/weekday							498 min(B)^38^, 503 min(G)^38^
Min or %/weekend day							455 min(B)^38^, 430 min(G)^38^
Min or %/school time							
Min or %/leisure time							
**Television time**							
Min or h/day	87 min^25^		142 min^25^				
Min or h/weekday		3.2-2.8-2.5 h(B)^50^, 2.8-2.8-2.6 h(G)^50^			2.5-2.4-2.1 h(B)^50^, 2.2-2.1-1.9 h(G)^50^	2.5-2.2-2.2 h(B)^50^, 2.4-2.1-2.0 h(G)^50^	2.3-2.1-2.2 h(B)^50^, 2.2-2.0-2.1 h(G)^50^
Min or h/weekend day		3.9-3.7-3.2 h(B)^50^, 3.7-3.7-3.4 h(G)^50^			3.3-3.2-2.9 h(B)^50^, 2.9-3.0-2.8 h(G)^50^	3.3-3.0-2.7 h(B)^50^, 3.2-2.8-2.5 h(G)^50^	3.2-2.8-3.0 h(B)^50^, 2.9-2.6-2.8 h(G)^50^
% >2 h/day	40(B)^45^, 52(G)^45^	49(B)^45^, 50(G)^45^	57(B)^45^, 56(G)^45^		39(B)^45^, 33(G)^45^	36(B)^45^, 33(G)^45^	
% >2 h/weekday	73(B)^51^, 75(G)^51^	63(B)^51^, 60(G)^51^	70(B)^51^, 69(G)^51^		59(B)^51^, 50(G)^51^	63(B)^51^, 59(G)^51^	70(B)^51^, 67(G)^51^
% >3 h/weekday		56^40^	40^40^			43^40^	37^40^
% <1 h/day, 1-3 h/day, 3-5 h/day, >5 h/day						12, 52, 26, 11(A Coruña)^58^; 13, 50, 27, 11(Asturias)^58^; 10, 44, 31, 16(Barcelona)^58^; 12, 47, 27, 14(Bilbao)^58^; 8, 41, 34, 17(Cartagena)^58^; 9, 45, 32, 14(Madrid)^58^; 14, 50, 25, 11(San Sebastian)^58^, 9, 47, 30, 15(Valencia)^58^; 8, 46, 31, 15(Valladolid)^58^	
% ≤0.5 h, 1-2 h, 3-4 h, >4 h/schoolday							
**Computer time**							
Min or h/day	15 min^25^		3 min^25^				
Min or h/weekday		1.7-2.8-4.3 h(B)^50^, 0.7-1.8-3.6 h(G)^50^			1.3-3.1-3.4 h(B)^50^, 0.6-1.9-2.5 h(B)^50^	1.1-2.2-3.2 h(B)^50^, 0.7-1.6-2.8 h(G)^50^	1.8-3.6-4.1 h(B)^50^, 0.9-2.3-3.1 h(G)^50^
Min or h/weekend day		2.4-3.8-5.6 h(B)^50^, 1.0-2.6-4.7 h(G)^50^			2.1-4.2-4.6 h(B)^50^, 1.1-2.7-3.3 h(G)^50^	1.8-3.7-4.1 h(B)^50^, 1.1-2.5-3.7 h(G)^50^	2.3-4.3-5.1 h(B)^50^, 1.1-2.7-3.7 h(G)^50^
% >1 h/day							
% >2 h/day	24(B)^45^, 16(G)^45^	12(B)^45^, 9(G)^45^	17(B)^45^, 13(G)^45^		17(B)^45^, 16(G)^45^	11(B)^45^, 10(G)^45^	
% >2 h/weekday	59^51^	30^40^, 70^51^	23^40^, 68^51^		58^51^	20^40^, 67^51^	32^40^, 74^51^
**Videogames time**							
Min or h/day	16 min^25^		23 min^25^				
% >2 h/day	45(B)^45^, 24(F)^45^	30(M)^45^, 13(F)^45^	35(B)^45^, 11(G)^45^		26(B)^45^, 6(G)^45^	16(B)^45^, 7(G)^45^	
% >2 h/weekday	46^51^	42^51^	47^51^		27^51^	39^51^	46^51^
**Total screen-time**							
Min or h/day	125 min(B)^23^, 111 min(G)^23^		118 min(B)^23^, 139 min(G)^23^				
Min or h/weekday	152 min(B)^23^, 120 min(G)^23^		252 min(B)^23^, 196 min(G)^23^				
**Total sedentary time**		**Switzerland**	**Macedonia**		**Turkey**	**Ukraine**	**UK**
Min or %/day							356 min^53^, 362 min^53^, 352 min^53^
Min or %/weekday							
Min or %/weekend day							
Min or %/school time							
Min or %/leisure time							
**Television time**							
Min or h/day							119 min^25^
Min or h/weekday		1.8-1.6-1.4 h(B)^50^, 1.7-1.4-1.3 h(G)^50^	2.8-2.5-2.4 h(B)^50^, 2.5-2.5-2.4 h(G)^50^			3.6-2.9-2.5 h(B)^50^, 3.3-3.0-2.6 h(G)^50^	2.9-2.7-2.5 h(B,SC)^50^, 2.8-2.5-2.3 h(G,SC)50, 2.9-2.6-2.6 h(B,WAL)^50^, 2.9-2.5-2.3 h(G,WAL)^50^
Min or h/weekend day		2.9-2.6-2.5 h(B)^50^, 2.6-2.4-2.4 h(G)^50^	3.5-3.5-3.1 h(B)^50^, 3.3-3.6-3.2 h(G)^50^			4.5-3.7-3.2 h(B)^50^, 4.5-4.0-3.3 h(G)^50^	3.4-3.2-3.2 h(B,SC)^50^, 3.2-2.8-2.9 h(G,SC)^50^ 3.4-3.2-3.2 h(B,WAL)^50^, 3.5-3.0-2.9 h(G,WAL)^50^
% >2 h/day		19(B)^45^, 17(G)^45^	44(B)^45^, 45(G)^45^		43(B)^45^, 41(G)^45^	54(B)^45^, 57(G)^45^	37(B, ENG)^45^, 31(G,ENG)^45^
% >2 h/weekday		58(B)^51^, 51(G)^51^	57(B)^51^, 56(G)^51^			61(B)^51^, 64(G)^51^	67(B,ENG)^51^, 66(G,ENG)^51^, 72(B,SC)^51^, 64(G,SC)^51^, 72(B,WAL)^51^, 73(G,WAL)^51^
% >3 h/weekday		24^40^	48^40^			66^40^	52(ENG)^40^, 50(SC)^40^, 53(WAL)^40^
% <1 h/day, 1-3 h/day, 3-5 h/day, >5 h/day			3, 34, 41, 23^58^				
% ≤0.5 h, 1-2 h, 3-4 h, >4 h/schoolday							22, 50, 20, 8(SC)^27^
**Computer time**							
Min or h/day							11 min^25^
Min or h/weekday		1.1-2.2-2.3 h(B)^50^, 0.6-1.4-1.8 h(G)^50^	1.4-3.0-3.4 h(B)^50^, 0.8-2.1-3.4 h(G)^50^			1.1-2.6-2.8 h(B)^50^, 0.4-1.2-2.1(G)^50^	2.1-3.9-4.5 h(B,SC)^50^, 1.2-2.8-3.5 h(G,SC)^50^, 1.7-3.6-4.2 h(B,WAL)^50^, 1.0-2.8-3.5 h(G,WAL)^50^
Min or h/weekend day		1.9-3.8-4.0 h(B)^50^, 1.0-2.4-3.1 h(G)^50^	2.0-4.9-6.0 h(B)^50^, 1.2-3.6-5.2 h(G)^50^			1.6-3.7-3.7 h(B)^50^, 0.5-1.8-2.9 h(G)^50^	2.5-4.6-5.6(B,SC)^50^, 1.3-3.2-4.2 h(G,SC)^50^, 2.2-4.4-5.1 h(B,WAL)^50^, 1.3-3.3-4.2 h(G,WAL)^50^
% >1 h/day							
% >2 h/day		12(B)^45^, 8(G)^45^	16(B)^45^, 13(G)^45^		18(B)^45^, 16(G)^45^	12(B)^45^, 5(G)^45^	25(B,ENG)^45^, 25(G,ENG)^45^
% >2 h/weekday		16^40^, 53^51^	26^40^, 55^51^			17^40^, 64^51^	37(ENG)^40^, 39(SC)^40^, 33(WAL)^40^, 72(ENG)^51^, 78(SC)^51^, 72(WAL)^51^
**Videogames time**							
Min or h/day							37 min^25^
% >2 h/day		11(B)^45^, 3(G)^45^	26(B)^45^, 12(G)^45^		22(B)^45^, 8(G)^45^	25(B)^45^, 8(G)^45^	25(B,ENG)^45^, 8(G,ENG)^45^
% >2 h/weekday		31^51^	36^51^			43^51^	45(ENG)^51^, 54(SC)^51^, 50(WAL)^51^
**Total screen-time**							
Min or h/day							
Min or h/weekday							

This table displays a summary of the results reported in the articles included in the systematic review; *B* boys, *G* girls, *min* minutes, *h* hours, *FL* Flemish part of Belgium, *FR* French part of Belgium, *ENG* England, *SC* Scotland, *WAL* Wales; references are displayed in superscript to avoid confusion with the levels of sedentary time

The data clearly show a large variation in reported outcome variables and assessment methods by article, which makes it difficult to describe the child and adolescent population levels. Despite this large variation, in general, higher values for sedentary time were observed in children and adolescents from more East-European countries as compared to the rest of Europe, especially for television viewing.

Further, large differences were observed between articles from the same country. One study illustrated the large differences that can be observed between assessment methods even within the same study, namely there were differences in television viewing, computer use and total screen-time recorded between the usual frequency and the 24 h-recall question type [28].

**Fig. 2:**
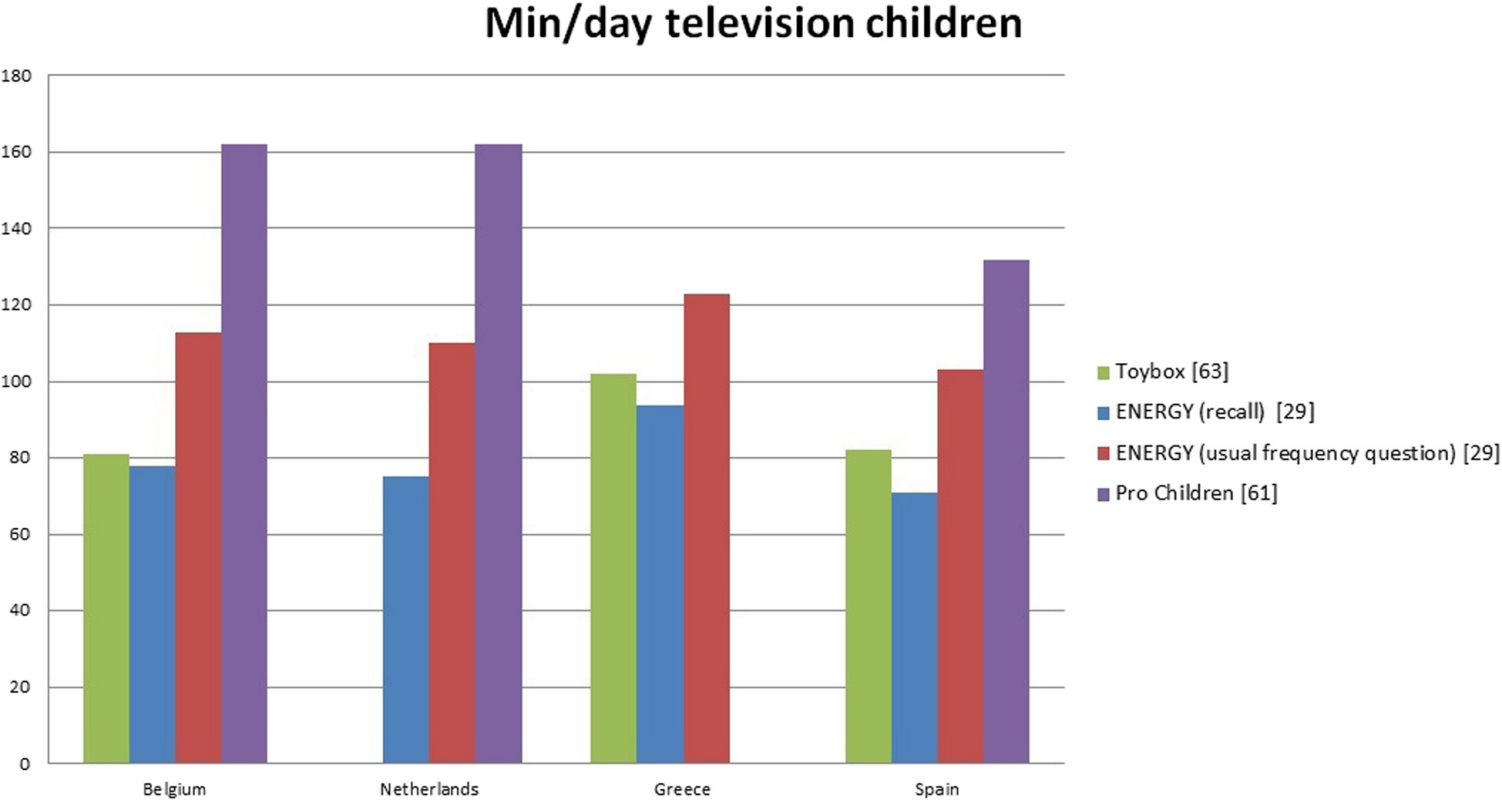
Minutes per day of television viewing in different articles for children from four European countries

To provide a more accessible overview of the results, the bar charts in Fig. 2 display the amount of minutes per day that children spent in watching television across four countries using different assessment methods. Three articles were available [28, 60, 62]: one article had data for the four countries [28] and two articles had data for three out of four countries [60, 62]. In one article [28], television time was assessed by both a usual frequency and 24 h-recall questionnaire. In the article using data from the Toybox study [60], we calculated minutes of television time per day by following formula: ((min/weekday*5) + (min/weekend day*2))/7. The highest levels of television time were observed within the article containing data from the Pro Children study (9-11-year-olds), followed by the article containing usual frequency questionnaire data from the ENERGY study (10-12-year-olds).

### Variation in assessment methods and reported sedentary time variables

Table 4 provides an overview of the assessment methods and sedentary time outcome variables used in the retrieved articles. To describe this variation, we have again included all eligible articles (*n* = 42), as articles from the same European study sometimes reported different outcome variables or reported the same outcome variable differently. Some articles used several assessment methods and/or reported several outcome variables. Most articles used a child questionnaire (*n* = 25), with others using accelerometers (*n* = 10). Interview with parents was conducted in one study, and in three other studies adolescents were asked to complete an ecological momentary assessment. Questionnaires were used to assess time spent in domains of sedentary time, whilst accelerometers were used to assess total sedentary time. With regard to the domains of sedentary time, television time was assessed in 24 articles, computer time in 15 articles, total screen-time in 11 articles and total sedentary time in 10 articles. Some articles described a specific time period, such as before (*n* = 1), during (*n* = 2) and after school hours (*n* = 3). The outcome variables were mostly expressed in minutes (*n* = 16) or hours (*n* = 11) over a specific time period or the percentage exceeding more than 2 h per day (*n* = 12).

**Table 4:** Assessment methods and reported outcome variables in the articles included in the systematic review

	Number	Reference number
Measurement		
ActiGraph accelerometer (100 cpm cut-point and 10 min non-wear time)	1	EYHS[36]
ActiGraph accelerometer (100 cpm cut-point and 20 min non-wear time)	6	ENERGY [31–33], EYHS [37], ISCOLE [58, 59]
ActiGraph accelerometer (100 cpm cut-point and 60 min non-wear time)	2	ICAD [52, 53]
ActiGraph accelerometer (500 cpm cut-point and 10 min non-wear time)	1	EYHS [34]
Self-administered child questionnaire	25	[25, 26], COSI [27], ENERGY [28–30], EYHS [35, 38], HBSC 01/02 [39–43], HBSC 05/06 [44–46], HBSC 09/10 [47–49], HBSC 13/14 [50], ICAD [51], ISAAC [57], ISCOLE [59], Pro Children [60, 61]
Self-administered parental questionnaire	7	ICAD [51], IDEFICS [54–56], ISAAC [57], Toybox [62, 63]
Parental questionnaire interview	1	Toybox [63]
Ecological Momentary Assessment Diary	3	[22–24]
Child and parental questionnaire: question type		
Usual frequency	28	[26], COSI [27], ENERGY [28–30], EYHS [35, 38], HBSC 01/02 [39–43], HBSC 05/06 [44–46], HBSC 09/10 [47–49], HBSC 13/14 [50], ICAD [51], IDEFICS [54–56], ISAAC [57], ISCOLE [59], Pro Children [60, 61], Toybox [62]
Recall	1	ENERGY [28]
Unknown	2	[25], Toybox [63]
Child and parental questionnaire: answer type		
Questions with answer categories	26	[26], COSI [27], ENERGY [28–30], EYHS [35, 38], HBSC 01/02 [39–43], HBSC 05/06 [44–46], HBSC 09/10 [47–49], HBSC 13/14 [50], IDEFICS [54–56], ISCOLE [59], Pro Children [60, 61], Toybox [62]
Questions without answer categories	-	-
Unknown	4	[25], ICAD [51], ISAAC [57], Toybox [63]
Reported specific sedentary time variable		
Total sedentary time	10	ENERGY [31–33], EYHS [34, 36, 37], ICAD [52, 53], ISCOLE [58, 59]
Television time	24	[23–26], COSI[27], ENERGY [28], EYHS [35, 38], HBSC 01/02 [39–43], HBSC 05/06 [44–46], HBSC 09/10 [48, 49], HBSC 13/14 [50], ISAAC [57], Pro Children [60, 61], Toybox [62, 63]
Computer time	15	[23, 24], COSI [27], ENERGY [28], EYHS [35, 38], HBSC 01/02 [39, 43], HBSC 05/06 [44–46], HBSC 09/10 [47, 49], HBSC 13/14 [50], Toybox [62]
Videogames time	6	[23, 24], HBSC 05/06 [44–46], HBSC 13/14 [50]
Screen-time	11	[22], COSI [27], ENERGY [28–30], ICAD [51], IDEFICS [54–56], ISCOLE [59], Toybox [62]
Homework	3	[23, 24], HBSC 01/02 [43]
Other sedentary activities	4	[22–24], Toybox [62]
Reported time period		
Day	28	[24, 25],COSI [27], ENERGY [28–30, 32], EYHS [34, 35, 37, 38], HBSC 01/02 [41, 42], HBSC 05/06 [44, 45], ICAD [51–53], IDEFICS [54–56], ISAAC [57], ISCOLE [58, 59], Pro Children [60, 61], Toybox [62, 63]
Weekday	14	[22, 23, 26], EYHS [36, 37], HBSC 01/02 [39, 40, 43], HBSC 05/06 [46], HBSC 09/10 [47–49], HBSC 13/14 [50], Toybox [62]
Weekend day	8	[22, 23], EYHS [36, 37], HBSC 01/02 [40, 43], HBSC 09/10 [49], Toybox [62]
School time	2	ENERGY [31], EYHS [36]
Before school	1	EYHS [38]
After school	3	EYHS [35, 36, 38]
Reported unit		
Minutes	16	[22–24], ENERGY [28–33], EYHS [36, 37], HBSC 09/10 [47], ICAD [52, 53], ISCOLE [58], Toybox [62]
Hours	11	COSI [27], EYHS [38], HBSC 01/02 [40, 42], HBSC 05/06 [45], HBSC 09/10 [47, 49], ISAAC [57], ISCOLE [59], Pro Children [60], Toybox [63]
% of time period	2	ENERGY [31], EYHS [34]
% >1 hour	5	EYHS [35, 38], IDEFICS [55, 56], Toybox [62]
% >2 hours	12	[25], EYHS [35, 38], HBSC 01/02 [39], HBSC 05/06 [44, 46], HBSC 09/10 [48], HBSC 13/14 [50], ICAD [51], IDEFICS [56], ISCOLE [59], Pro Children [61]
% >3 hours	2	HBSC 01/02 [39, 43]
% >4 hours	2	HBSC 01/02 [41, 43]
% not at all, <0.5 h, 0.5-1 h, 1-2 h, 2-3 h, >3 h	1	IDEFICS [54]
% <0.5 h, 1-2 h, 3-4 h, >4 h	1	[26]
% <1 h, 1-3 h, 3-5 h, >5 h	1	ISAAC [57]

*h* hours, *COSI* WHO European Childhood Obesity Surveillance Initiative, *ENERGY* EuropeaN Energy balance Research to prevent excessive weight Gain among Youth, *EYHS* European Youth Heart Study, *HBSC* Health Behaviour in School-aged Children, *ICAD* International Children’s Accelerometer Database, *IDEFICS* Identification and prevention of Dietary and lifestyle induced health Effects In Children and infantS, *ISAAC* International Study of Asthma and Allergies in Childhood, *ISCOLE* The International Study of Childhood Obesity, Lifestyle and the Environment

## Discussion

This systematic review aimed to provide an overview of existing cross-European studies assessing sedentary time in children (0-12y) and adolescents (13-18y), to describe the variation in population levels of sedentary time and to discuss the impact of assessment methods.

### Overview of existing cross-European studies

The literature search revealed 42 articles reporting on levels of sedentary time. Thus, the current systematic review included the highest number of eligible articles in comparison with the other three reviews on sedentary time in adults and on physical activity in youth and adults [16–18]. Although sedentary time has only received increased attention in the last few years, earlier studies have described children and adolescents’ television and screen-time [64].

Nine articles that were part of the HBSC-studies included the most countries (up to 36), but there were still some countries for which no data were available in cross-European studies. These countries should therefore be included in further European surveillance studies in order to have a complete overview of the sedentary time levels among children and adolescents. Since 38 of 42 articles were cross-sectional, future longitudinal studies could shed light on how sedentary time varies over time in the same population of children and adolescents. However, conducting repeated cross-sectional studies is also of importance in terms of public health to understand trends in sedentary time.

### Variation in population levels of sedentary time and impact of assessment methods

The tables with data on the levels of sedentary time in children and adolescents across European countries might be useful for European researchers and policy makers, as they provide an orderly reference work of conducted cross-European studies. One general conclusion that we might draw from the results is that children and adolescents from Eastern-European countries (i.e. the more eastern part of Europe such as Bulgaria, Slovakia, Ukraine) have somewhat higher levels of sedentary time compared to the rest of Europe. However, there are several plausible reasons for the large differences observed between articles. First, different assessment methods were used. Child-specific questionnaires were used in 60 % of the articles and were only designed to measure time spent in domain-specific sedentary activities. Accelerometers were the only assessment methods that measured the total sitting time and were used in 24 % of the articles, probably because greater cost incurred in using accelerometers in large-scale studies. However, as technological advances have made the accelerometers smaller, lighter, and less expensive, it has been argued that the accelerometer has now become feasible for use in large-scale studies. An important remark is that standard procedures to process accelerometer data are then needed [65]. To estimate children’s total sedentary time via accelerometers, sedentary time was measured by summing the recorded epochs during which the average accelerometer counts were equivalent to less than 100 counts per minute, which is the most commonly used threshold for sedentary time measurement [66, 67]. Another assessment method that might also be less feasible to use in large-scale studies is the ecological momentary assessment tool. This method was used in three cross-European articles, but included a rather limited number of participants and countries, as this assessment method brings along a high time burden for participants. Next to variation in assessment methods, the included articles also reported different outcome variables (e.g. television time vs. total screen-time) or reported the same outcome variable differently (e.g. television time expressed in minutes per day vs. expressed in the percentage exceeding the 2 h recommendation). Finally, the amount of sedentary time was observed to substantially vary in individual countries across different articles. Among Estonian female adolescents for example, total sedentary time on a weekday was less than six hours in one article [36] and almost nine hours in another article [37]. These differences might have emerged because of age differences between study samples. In this review, separate tables were designed for children and adolescents, but age differences can still cause the differences in population levels between and within countries, as the amount of sedentary time increases with age [68]. Thus, because of these large methodological differences between studies, we want to emphasise that cross-European comparisons are currently only possible within studies.

### Limitations and strengths

This review has some limitations that should be acknowledged. A first limitation is that although the search was performed in several databases in combination with multiple additional search strategies (e.g. back- and forward tracking), there is still a possibility that not all existing studies on this topic were covered. Some articles might not be found in our databases searched or through our search strategy. The use of including only English published data might also contribute to this limitation, although we expect that results of cross-European studies would be published in English. Another possible limitation could be that only cross-European studies were included. Single-country studies may have provided additional information. However, the purpose was to specifically review the literature on cross-country studies so that the results across countries would at least be comparable within articles [15]. This also means that cross-European studies that did not report the outcome separately per country were excluded in the review, such as the HELENA (Healthy lifestyle in Europe by nutrition in adolescence) study [69]. An important strength is the systematic process: there was a written protocol for all four reviews that was agreed upon by all involved researchers and the search, article selection, data extraction and quality assessment were conducted together for all four reviews. Also, each step of the review process has been conducted by two independent researchers with issues being resolved by consulting a third researcher.

### Recommendations for the future

This systematic literature review showed that there is a need for harmonisation and standardisation of methods to assess sedentary time in European children and adolescents. The same conclusion was drawn from the other systematic reviews conducted within DEDIPAC for sedentary time in adults and for physical activity in youth and adults [16–18]. A possible approach for the future could be to add objective assessment methods in existing large cross-European surveillance systems, such as the HBSC-study. Another approach could be to conduct a pooled analysis on existing data of European children and adolescents (and adults). This is similar as the approach of the International children’s accelerometry database (ICAD) which collected, pooled and reduced individual accelerometer data files using standardised methods to compare the outcome variables across studies [70]. However, it might be difficult to obtain accelerometer data from all European countries, as few countries have population representative accelerometer data. Conducting a pooled analysis on existing questionnaire data would also be difficult, as harmonisation of data from different questionnaires is even more challenging. A final approach could be to set up a new cross-European surveillance system combining objective and self-report methods (for example, accelerometers and questionnaires) to monitor levels of sedentary time and physical activity in children, adolescents and adults.

## Conclusion

Generally, higher levels of sedentary time were observed in children and adolescents from Eastern-European countries. There was a large variation in assessment methods and outcome variables across cross-European studies. Questionnaires (child specific) were used most often, probably because of feasibility reasons. These self-report measures mostly measured screen-based activities only, rather than total sedentary time. In sum, to enable cross-European surveillance, there is a need for harmonisation and standardisation of methods to assess sedentary time in European children and adolescents. Such a surveillance system should combine objective and self-report methods.

## Abbreviations

B, boys; COSI, WHO European childhood obesity surveillance initiative; ENERGY, EuropeaN energy balance research to prevent excessive weight gain among youth; ENG, England; EYHS, European youth heart study; FG, usual frequency question; FL, Flemish part of Belgium; FR, French part of Belgium; G, girls; h, hour; HBSC, health behaviour in school-aged children; ICAD, International Children’s Accelerometer Database; IDEFICS, identification and prevention of dietary and lifestyle induced health Effects In Children and infantS; ISAAC, International Study of Asthma and Allergies in Childhood; ISCOLE, The International Study of Childhood Obesity, Lifestyle and the Environment; min, minutes; SC, Scotland; SES, socio-economic status; WAL, Wales

## Additional files

Additional file 1: PRISMA 2009 Checklist. (PDF 195 kb) Additional file 2: Search strategy. (PDF 175 kb) Additional file 3: Data extraction file. (XLSX 80 kb) Additional file 4: Quality assessment file. (PDF 252 kb)
