# Supplementary material for: Variation in population levels of sedentary time in European children and adolescents according to cross-European studies: a systematic literature review within DEDIPAC
Source: Int J Behav Nutr Phys Act. 2016 Jun 28;13:69. doi: 10.1186/s12966-016-0395-5 (PMC4924322; doi:10.1186/s12966-016-0395-5)
Supplement: Additional file 4: — Quality assessment file. (PDF 252 kb) [file 12966_2016_395_MOESM4_ESM.pdf]

Additional file 4: Quality assessment file

**Publication**	**1. Objective**	**2. Study design**	**3. Subject selection**	**4. Subject characteristics**	**8. Outcome measure(s)**	**9. Sample size**	**10. Analytic methods**	**11. Estimate of variance**	**12. Confounding**	**13. Results**	**14. Conclusions**	**Score**
Biddle et al. (2009) [23]	2	2	2	2	2	1	2	2	1	2	2	**0.91**
Soos et al. (2012) [24]	1	2	2	2	1	1	2	2	0	2	2	**0.77**
Soos et al. (2014) [25]	2	2	1	1	2	1	2	2	2	2	2	**0.86**
Cinar & Murtomaa (2008) [26]	2	2	1	2	2	2	2	2	2	2	2	**0.95**
Hanewinkel et al. (2012) [27]	1	2	1	2	2	1	N/A	2	N/A	2	2	**0.83**
Börnhorst et al. (2015) [28]	1	2	2	2	2	2	2	2	2	2	2	**0.95**
Brug et al. (2012) [29]	2	2	2	2	2	2	2	2	2	2	2	**1.00**
Brug et al. (2012) [30]	2	2	2	2	1	1	2	2	2	2	2	**0.91**
Fernandez-Alvira et al. (2013) [31]	2	2	2	2	1	2	2	2	2	2	2	**0.95**
van Stralen et al. (2013) [32]	2	2	2	2	2	1	2	2	2	2	2	**0.95**
Verloigne et al. (2012) [33]	2	2	2	2	2	1	2	2	2	2	2	**0.95**
Yildirim et al. (2014) [34]	2	2	2	2	2	1	2	2	2	2	2	**0.95**
Ekelund et al. (2004) [35]	2	2	2	2	2	2	2	2	2	2	2	**1.00**
Jago et al. (2008) [36]	2	2	1	2	2	2	2	2	2	2	2	**0.95**
Nilsson et al. (2009) [37]	2	2	2	2	2	2	2	2	2	2	2	**1.00**
Ortega et al. (2013) [38]	2	2	1	2	2	1	2	2	2	2	2	**0.91**
van Sluijs et al. (2008)	2	2	2	2	1	2	2	2	2	2	2	**0.95**

[39]												
Janssen et al. (2005) [40]	2	2	2	1	2	2	2	2	2	2	2	**0.95**
Kuntsche et al. (2006) [41]	1	2	2	1	2	2	2	2	2	2	2	**0.91**
Richter et al. (2009) [42]	2	2	2	1	2	2	2	2	2	2	2	**0.95**
Vereecken et al. (2006) [43]	2	2	2	0	2	2	2	2	2	2	2	**0.91**
HBSC report 2004 [44]	1	2	2	2	2	2	1	0	0	2	2	**0.73**
Haug et al. (2009) [45]	2	2	2	2	2	2	2	2	2	2	2	**1.00**
Torsheim et al. (2010) [46]	2	2	2	0	2	2	2	2	2	2	2	**0.91**
HBSC report 2008 [47]	1	2	2	2	2	2	1	0	0	1	2	**0.68**
Nuutinen et al. (2015) [48]	2	2	2	2	2	2	2	2	2	2	2	**1.00**
HBSC report 2012 [49]	1	2	2	2	2	2	1	0	0	1	2	**0.68**
Bucksch et al. (2016) [50]	2	2	1	1	2	2	2	0	2	2	2	**0.82**
HBSC report 2016 [51]	1	2	2	2	1	2	1	0	0	1	2	**0.64**
Atkin et al. (2014) [52]	2	1	1	1	1	2	2	2	2	2	2	**0.82**
Ekelund et al. (2012) [53]	2	1	1	2	2	2	2	2	2	2	2	**0.91**
Hildebrand et al. (2015) [54]	1	2	1	2	2	2	2	2	2	2	2	**0.91**
Hense et al. (2011) [55]	2	2	2	2	0	2	2	2	2	2	2	**0.91**
Hunsberger et al. (2012) [56]	2	2	1	2	0	2	2	2	2	2	2	**0.86**
Kovàcs et al. (2015) [57]	2	2	2	1	2	2	2	2	2	2	2	**0.95**
Mitchell et al. (2013) [58]	1	2	1	1	2	2	2	2	2	2	2	**0.86**
Katzmaryk et al. (2015) [59]	2	2	2	2	2	2	2	2	1	2	2	**0.95**

LeBlanc et al. (2015) [60]	2	2	1	2	2	2	2	2	2	2	2	**0.95**
Klepp et al. (2007) [61]	2	2	2	2	2	2	2	2	2	2	2	**1.00**
te Velde et al. (2007) [62]	2	2	2	1	2	2	2	2	2	2	2	**0.95**
De Craemer et al. (2015) [63]	2	2	2	1	2	2	2	2	2	2	2	**0.95**
van Stralen et al. (2012) [64]	2	2	1	2	1	2	2	2	2	2	2	**0.91**
**Total**	**74**	**82**	**71**	**70**	**73**	**75**	**78**	**74**	**70**	**81**	**84**	**0.91**

Since items 5 (random allocation), 6 (blinding investigators) and 7 (blinding subjects) were not applicable to all of the studies, they are not shown here; N/A, not applicable
